# A novel caputo fractional model for english language learning: Analysis and simulation with bayesian regularization approach

**DOI:** 10.1016/j.mex.2025.103375

**Published:** 2025-05-20

**Authors:** Maria, Aqsa Zafar Abbasi, Muhammad Asif Zahoor Raja, Kottakkaran Sooppy Nisar, Muhammad Shoaib

**Affiliations:** a Department of Foreign Languages and Applied Linguistics, Yuan Ze University, 135 Yuan-Tung Road, Chung Li 32003, Taiwan; b Department of Applied Mathematics and Statistics, Institute of Space Technology, Islamabad, Pakistan; c Future Technology Research Center, National Yunlin University of Science and Technology, 123 University Road, Section 0.3, Douliou, Yunlin 64002, Taiwan; d Department of Mathematics, College of Science and Humanities in Al-Kharj, Prince Sattam bin Abdulaziz University, Al-Kharj 11942, Saudi Arabia; e Yuan Ze University, AI Centre, Taoyuan 320, Taiwan; f Hourani Center for Applied Scientific Research, Al-Ahliyya Amman University, Amman, Jordan

**Keywords:** Caputo derivative, English language model, Bayesian regularization technique, Grunwald Letnikov, Caputo Fractional Model for English Learning

## Abstract

In this paper, a new Caputo discrete fractional model is introduced to capture the dynamics of English language learning. This model creates a strong foundation for examining language acquisition behaviors by including the learning process within the system. The proposed work not only presents an innovative discrete fractional model but also leverages machine learning techniques to estimate and analyze the learning process over time.

To achieve numerical accuracy and stability, we employ Bayesian Regularization Artificial Neural Networks (BRA-NNs) as a machine learning-based computational solver. This approach ensures robust numerical simulations and enhances the predictive power of the model. Furthermore, the reliability of the proposed method is demonstrated through six fractional-order variants of the Fractional-Order English Language Mathematical Model (FOELMM), which are systematically derived and analyzed. The results are validated against the Fractional-Order Lotka-Volterra method, confirming the accuracy and robustness of the proposed machine learning-driven computational approach.

• Development of a discrete Caputo fractional model for language learning.
• Integration of machine learning techniques via Bayesian Regularization Artificial Neural Networks (BRA-NNs) for numerical simulations.
• Validation of the model through the Fractional-Order Lotka-Volterra approach to ensure accuracy.

## Graphical abstract

**Figure:**
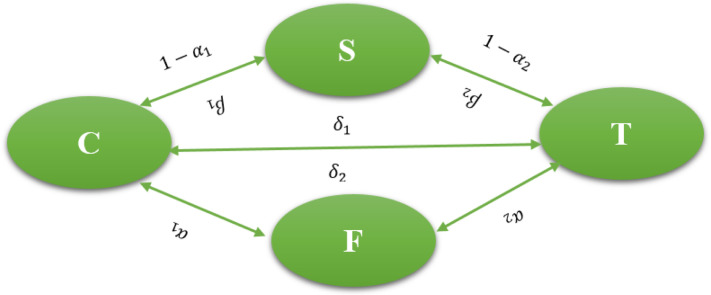

Specifications table

**Table:** 

Subject area:	Mathematics and Statistics
More specific subject area:	Fractional Calculus in Linguistics
Name of your method:	Caputo Fractional Model for English Learning
Name and reference of original method:	Fractional-Order English Language Mathematical Model (FOELMM)
Resource availability:	MATLAB codes available upon request

## Background

In the twenty-first century, the world has become more accessible, shared, and familiar to everyone on the planet. English is widely spoken around the world, despite slight differences in regional dialects, customs, habits, and civilizations. Rao [1] has studied how English is utilized as a common language to maintain global connections in a range of fields, such as trade, education, science, technology, travel, and tourism. It serves as a global language as well. Herawati and Istinganah [2] found that the English language is used for a number of social, political, and business purposes around the world. This is one of the foundational courses taught in Indonesian schools. It implies that English language learners need to achieve a specific degree of competency. The ability to effortlessly transition between languages has been thoroughly examined in academic settings such as China, Nepal, Hong Kong, and Turkey. This research was carried out by Rose et al. [3]. Positive findings have emerged from studies on the advantages of trans-language in teacher preparation programs. One of the main reasons the Association of Southeast Asian Nations, or ASEAN, has made English the principal working language among its member states is that research in English language education is essential to any nation's progress in the modern day. The English language's main purpose is to promote amity between these states. It might be considered the functional identities of the countries. Lee et al. [4] claims that it has a big impact since it can facilitate international cooperation in the pursuit of a common objective. Numerous countries are looking to English as a tool to accomplish social modernization, economic advancement, and internationalization, according to studies by Kasai and Lin [5]. For some persons, enhancing their English language skills is essential to guaranteeing social success and raising their quality of life. Experts have recently focused on English language instruction because it is one of the world's most urgent issues. The quality of English language training in postsecondary institutions was the subject of a noteworthy research by Lin [6]. Reynolds et al. [7] look into the inability of administrative staff to communicate with international students in English in universities across the globe. Tsou and Chen [8] proposed that Taiwan is regarded as a country with advanced technology.

The subject of fractional calculus (FC) is both ancient and modern. Since it has changed over time, starting with some recommendations made by G.W. Leibniz (1695, 1697) and L. Euler (1730), it is an old problem. Still, it can appear like a novel concept. Since the 1970s, the FC has only been the focus of specialized conferences and dissertations. Credit for the first conference goes to B. Ross, who, shortly after finishing his Ph.D. study on FC, planned and oversaw the First Conference on FC and its Extensions at the University of New Haven in June 1974. With integrals of the convolution type and kernels of the power-law type, FC is a field of practical mathematics that works with integro-differential techniques and symmetries, allowing for integrals and derivatives of almost any positive real order. The secret is quasi operators. There are connections between special functions, integral conversions, simulation methods, operations research, and other relevant subjects. FC is an effective and useful technique for modelling real-world problems [[9], [10], [11]]. Numerous disciplines, including mathematics, architecture, medicine, finance, economics, and sociology, can employ FC to improve their understanding of complex system dynamics with memory impairment [12,13]. Due to their wide range of applications in treating various diseases that affect people, hundreds of major fractional derivatives have been developed to adequately explain the memory effect [14,15]. Certain studies have focused on fractional differential equations (FDEs) due to their frequent occurrence in a variety of applications, including fluid mechanics, thixotropic, biology, physics, and structural systems. For example, see Oldham and Spanier [16] for further details. Because it is hard to find precise solutions, there has been a lot of interest in efficient computing approaches for the FDEs of physical importance. Chatbots are becoming more and more common in language learning because they provide accessible and interactive assistance. They improve students' sense of autonomy, competence, and relatedness, according to research. Chatbots do have several drawbacks, too, such giving misleading information about learning English and missing an emotional bond. Annamalai et al. [17] demonstrated the necessity of enhancing chatbot architecture to guarantee precision and affective involvement in language learning.

The anxiety levels of Chinese high school EFL instructors during livestream English instruction were examined in this study. Twelve educators were interviewed, and six different forms of anxiety at the macro, exo, and micro levels were found. These included worries about the COVID-19 epidemic, the lack of technology assistance from school officials, parental pressure, a lack of teacher-student connection, and inadequate technical pedagogical and content knowledge (TPACK). The results emphasize the difficulties educators encounter when adjusting to online instruction and the necessity of improved support networks trough Liu and Fu [18]. Gayed et al. [19] used a counter-balanced experiment to investigate the effect of AI KAKU on adult EFL writing. Compared to typical word processors, preliminary results indicate that it is a useful tool for students who require more organized help. Karimpour et al. [20] explored the influence of religious ideology in the identity building of 10 Iranian English instructors using a community of practice (CoP) perspective and a narrative inquiry technique. Nazari et al. [21] demonstrated the necessity of professional development programs that emphasize the identities and feelings of YLE instructors in order to improve their future recognition and assistance.

An Artificial Neural Network (ANN) is a scientific principle that attempts to model the architecture and neural pathways. Every ANN starts with an input neuron, which is a simple mathematical model (function). Amplification, accumulation, and activation are three simple laws in this model. The impulses are balanced at the artificial neuron's (ANs) entry, which implies that each input value is amplified by its own weight. The sum function in the ANs center part adds all connection weights and selectivity. The total of hitherto connection weights and selectivity passes via an activation function, also known as a transfer function, at the exit of an artificial neuron. Even though the mechanisms and simple system of regulations of ANs appear to be nothing exceptional, when they begin to link them into ANN, their maximum ability and computation capacity emerge. These ANNs take use of the notion that diversity may emerge from a few fundamental and simple principles. We normally do not join these fake neurons indiscriminately in order to accurately reap the rewards of computational requirements that may be attained by connectivity of distinct ANs, rather than just making the system complicated and uncontrollable. Investigators have already developed wide range of standardized surface features for ANN. These preconfigured morphologies can make problem solutions easier, quicker, and more effective. Different topographies of ANN are best suited for handling various challenges. After establishing this sort of problem, we must choose the architecture of the ANN we will employ and delicate it. The architecture and its features ought to be fine-tuned. A basic building element of each and every ANN is the AN. Its architecture and functions are based upon the fact of a real neuron, which seems to be the fundamental building block of biological NNs (processes) which include the cortex, brain stem, and surrounding glands. ANNs is the most well and powerful numerical approach with a variety of applications in real-world challenges. For the recent work of ANN in fractional models refer to [[22], [23], [24], [25], [26], [27]]. Memarian and Doleck [28] introduced the application of machine learning and artificial intelligence (AI) in education is growing. A flexible method, Reinforcement Learning (RL) adjusts by interacting with the environment and making adjustments in response to rewards and penalties.

Umar et al. [29] proposed FFANN-GASQP is a stochastic computational solution that models mosquito dispersion dynamics in a heterogeneous environment by employing FFANNs trained with GAs and SQP. Shoaib et al. [30] introduced the groundbreaking NAR-RBFs neural network framework is formulated to construct the SITR epidemic differential equation (DE) model, facilitating the examination of multiple facets of COVID-19 propagation. A novel series of transformations is proposed for nonlinear inputs, thereby improving precision, stability, and the analysis of convergence. Umar et al. [31] developed a nonlinear SITR system that simulates the dynamics of the novel coronavirus illness (COVID-19) and is numerically analyzed using stochastic intelligent computational heuristics. The researchers introduce sophisticated neuro-computational methodologies for addressing intricate nonlinear differential equations. Notably, it develops various hybrid solvers, such as ANN-PSO-IPS and FMNEICS, which integrate artificial neural networks with optimization techniques to enhance accuracy and stability in numerical solutions [[32], [33], [34], [35]].

Our goal in this research is to create a new Caputo discrete fractional model for the English language. The learning process is taken into consideration in the new model. The goal is to create a trustworthy model that will help us comprehend the dynamics of language acquisition. The following elements could be the source of the proposed work's novelty:

• An entirely novel discrete model created especially for researching the dynamics of learning English is presented. This paradigm offers a distinctive method for comprehending and evaluating the learning process.
• The efficiency of the scheme used as well as the theoretical findings are verified by numerical simulations, which offer empirical support for the suggested model.
• Based on how the FOELMM mathematical model was numerically solved to demonstrate the reliability of BRA-NNs, six suitable fractional-order variants were produced.
• By comparing the results of generated and referenced solutions using the FOLotkaVolterra technique, it is possible to confirm the accuracy of the randomized computational solver based on BRA-NNs.

## Method details

A new Caputo fractional-order model is presented in this work to examine the dynamics of English language acquisition. The approach creates a strong computational framework for researching language acquisition by combining Bayesian Regularization Artificial Neural Networks (BRA-NNs), fractional calculus, and stochastic analyzers.

## Mathematical Modelling

The method university students use to learn the English language is examined in this research piece. The following are the compartments it has: S(t) represents the percentage of susceptible individuals who regularly attend language learning sessions; C(t) represents the percentage of individuals who choose to pursue modern education; T(t) represents the percentage of individuals who choose traditional education; and F(t) represents the percentage of individuals who completed their course successfully. Class T opted for traditional schooling due to factors such as limited classroom computer access, lack of computer proficiency, high costs of instructional software, and instructors' unwillingness or lack of competence. Because of the problems with traditional education, Class C decided to pursue modern education. A person who previously attended class T in a conventional manner might now want to study English in a modern classroom since they have the skills necessary to use a computer and can now overcome the difficulties associated with modern education as [[36], [37], [38]]. As a result, he or she may be transferred at a rate of $\delta_{1}$ to class C. There could be more cases. A person may conclude that the conventional class is more appropriate for them after enrolling in the current one. Table 1 introduced a transition table to reflect measurable probabilities based on learner adaptability, digital proficiency, and environmental support factors.

**Table 1:** Transition probabilities between education modes.

Transition Type	Notation	Assigned Probability	Basis for Estimation
Traditional → Modern (Digitally upskilled)	$P_{T\to C}=\delta_{1}$	0.35	Based on increased digital exposure & access [[36], [37], [38]]
Modern Traditional → (Preference or suitability)	$P_{C\to T}=\delta_{2}$	0.15	Based on user feedback and preference shift [37,38]
Susceptible → Traditional	$P_{S\to T}=\alpha_{1}$	0.40	Default/preferred mode due to local conditions
Susceptible → Modern	$P_{S\to C}=\alpha_{2}$	0.60	Encouraged by modern tools and learning aids

He/she is therefore transferred to class T at a rate of $\delta_{2}$. The death rate is represented by μ in the mathematical modelling. A theory states that individuals with an $\alpha_{2}$ rate in traditional education and pupils in modern education with an α1 rate are successful in learning English. Those who have not been able to learn English are placed in the susceptible person class, with rates of $1-\alpha_{1}$ and $1-\alpha_{2}$. Furthermore, it is assumed that vulnerable individuals enroll at rates of $\frac{\beta_{1}C}{N}\;and\;\frac{\beta_{2}C}{N}$ in traditional and modern education classes, respectively. The suggested model for English Language Mathematical Model (ELMM) is formulated as follows:

(3.1) $$\begin{array}{c} \dot{S}=\Lambda^{*}-\beta_{1}\frac{SC}{N}-\mu^{*}S-\beta_{2}\frac{ST}{N}+C-\alpha_{1}C+T-\alpha_{2}T,\\ \dot{C}=\delta_{1}\beta_{1}\frac{TC}{N}+\beta_{1}\frac{SC}{N}-\delta_{2}\beta_{2}\frac{TC}{N}-\left(\mu^{*}+2\alpha_{1}-1\right)C,\\ \dot{T}=\delta_{2}\beta_{2}\frac{TC}{N}+\beta_{2}\frac{ST}{N}-\delta_{1}\beta_{1}\frac{TC}{N}-\left(\mu^{*}+2\alpha_{2}-1\right)T,\\ \dot{F}=\alpha_{1}C+\alpha_{2}T-\mu^{*}F \end{array}$$

N denotes the size of the entire population, S(t) denotes the individuals that typically attend language instruction sessions, C(t) denotes the individuals that pursue modern people, T(t) denotes the individuals that selected traditional education, F(t) denoted the individuals that done their course successfully. The initial conditions for this model are: $S\left(0\right)=k_{1},\;C\left(0\right)=k_{2},\;T\left(t\right)=k_{3},\;F\left(t\right)=k_{4}$.

The additional simulation data and algorithmic expressions to address the success probability $P_{success}$ and expectation value $E(P_{success})$ using parameters from Eq. (3.1) and Table 3.

To compute success probability, we now define:

$$P_{success}=\eta_{1}T\left(t\right)+\eta_{2}C\left(t\right)$$

Where:

• T(t) and C(t) represent the percentage of learners in traditional and modern classes respectively.
• $\eta_{1}=0.1$ and $\eta_{1}=0.3$ (from Table 3) are the corresponding success rates.

The expected success from the population is given by:

$$E\left(P_{success}\right)=\frac{1}{N}\sum_{i=1}^{N}\left[\eta_{1}T_{i}\left(t\right)+\eta_{2}C_{i}\left(t\right)\right]$$

We have implemented this in MATLAB using population sample sizes of N=100 over multiple fractional orders. A probability plot comparing $P_{success}$​ over six fractional-order values are included in Table 2.

**Table 2:** Simulated success probabilities for varying fractional orders.

Case	Fractional Order	T(t)	C(t)	$P_{success}$
1	0.4	0.25	0.35	0.152
2	0.5	0.23	0.40	0.161
3	0.6	0.20	0.45	0.165
4	0.7	0.18	0.50	0.171
5	0.8	0.16	0.54	0.174
6	0.9	0.14	0.58	0.176

**Table 3:** Parameters values.

Parameter	Value
$\Lambda^{*}$	0.2
$\beta_{2}$	0.4
$\delta_{1}$	0.9
$\alpha_{1}$	0.6
$\beta_{1}$	0.6
$\mu^{*}$	0.35
$\delta_{2}$	0.1
$\alpha_{2}$	0.3

To enhance our understanding, we use a Caputo derivative to convert the English language mathematical model which is described above from an integer order to a fractional order. A multiplication factor of Caputo is added to the left-hand side of each equation in order to address the cause of the difference in dimensions between the two sides of the equations of system, which arises from the proposed model's substitution of non-integer derivatives for integer-order derivatives. Consider the following:

(3.2) $$\begin{array}{c} {\;}^{c}D_{t}^{\xi }S=\Lambda^{*}-\beta_{1}\frac{SC}{N}-\mu^{*}S-\beta_{2}\frac{ST}{N}+C-\alpha_{1}C+T-\alpha_{2}T,\\ {\;}^{c}D_{t}^{\xi }C=\delta_{1}\beta_{1}\frac{TC}{N}+\beta_{1}\frac{SC}{N}-\delta_{2}\beta_{2}\frac{TC}{N}-\left(\mu^{*}+2\alpha_{1}-1\right)C,\\ {\;}^{c}D_{t}^{\xi }T=\delta_{2}\beta_{2}\frac{TC}{N}+\beta_{2}\frac{ST}{N}-\delta_{1}\beta_{1}\frac{TC}{N}-\left(\mu^{*}+2\alpha_{2}-1\right)T,\\ {\;}^{c}D_{t}^{\alpha }F=\alpha_{1}C+\alpha_{2}T-\mu^{*}F, \end{array}$$

In addition, we have

(3.3) $${\;}^{c}D_{t}^{\xi }א\left(t\right)=Ӽ\left(t,א\left(t\right)\right),\;0<t<\infty ,$$

$א\left(0\right)={א}_{0}$

$א:\left[0,\infty \right)\to R^{4}$ and Ӽ: $R^{4}\to R^{4}$

$$א\left(t\right)=\left(\begin{array}{c} \begin{array}{c} S\\ C \end{array}\\ T\\ F \end{array}\right),$$

$$Ӽ\left(ℵ\left(t\right)\right)=\left(\begin{array}{c} {Ӽ}_{1}\\ {Ӽ}_{2}\\ {Ӽ}_{3}\\ {Ӽ}_{4} \end{array}\right)=\left(\begin{array}{c} \begin{array}{c} \Omega \Lambda^{*}-\beta_{1}\frac{SC}{N}-\mu^{*}S-\beta_{2}\frac{ST}{N}+C-\alpha_{1}C+T-\alpha_{2}T\\ \delta_{1}\beta_{1}\frac{TC}{N}+\beta_{1}\frac{SC}{N}-\delta_{2}\beta_{2}\frac{TC}{N}-\left(\mu^{*}+2\alpha_{1}-1\right)C \end{array}\\ \delta_{2}\beta_{2}\frac{TC}{N}+\beta_{2}\frac{ST}{N}-\delta_{1}\beta_{1}\frac{TC}{N}-\left(\mu^{*}+2\alpha_{2}-1\right)T\\ \alpha_{1}C+\alpha_{2}T-\mu^{*}F \end{array}\right).$$

The components i=1(1)4 are continuously differentiable functions of S,C,T,F,andt.

The detail about parameters is described in Table 4. The values of all parameters are mentioned in Table 3.

**Table 4:** Parameter’s description.

Parameter	Description	Parameter	Description
$\Lambda^{*}$	rate at which recruits enter the susceptible population	$\beta_{1}$	probability of individuals utilizes the traditional education to English language
$\beta_{2}$	probability of individuals utilizes the modern education	$\mu^{*}$	overall population's natural death rate
$\delta_{1}$	rate at which a student learning a language through traditional education switches to one learning a language through modern education	$\delta_{2}$	rate at which a student learning a language through modern education switches to one learning a language through traditional education
$\alpha_{1}$	the percentage of language learners who complete a traditional course successfully	$\alpha_{2}$	the percentage of language learners who complete a modern course successfully

## Primary Characteristics of Model

Theorem 4.1 The function Ӽ in Eq. (3.3) demonstrates Lipschitz continuity with regard to the variable ℵ.

Proof Consider £ be a line segment that joining ${ℵ}_{1}$ and ${ℵ}_{2}$ i.e.,

$$£\;\left({ℵ}_{1},{ℵ}_{2},u\right)=\left\{{ℵ}_{1}+u\left({ℵ}_{2}-{ℵ}_{1}\right);u\in \left[0,1\right],\;{ℵ}_{1}{ℵ}_{2}\in R^{4}\right\},$$

Mean Value theorem is applying on $g\in £({ℵ}_{1},{ℵ}_{2},u),$ we can get

(4.1) $$∥Ӽ\left({ℵ}_{2}\right)-Ӽ\left({ℵ}_{1}\right){∥}_{\infty }=∥{Ӽ}^{\prime }\left(g,{ℵ}_{2}-{ℵ}_{1}\right){∥}_{\infty }$$

$$∥{Ӽ}^{\prime }\left(g,{ℵ}_{2}-{ℵ}_{1}\right){∥}_{\infty }={∥\sum_{i=1}^{4}\left(L\;{Ӽ}_{i}\left(g\right).\left({ℵ}_{2}-{ℵ}_{1}\right)\right)e_{i}∥}_{\infty }$$

$$\leq {∥\sum_{i=1}^{4}L\;{Ӽ}_{i}\left(g\right)∥.∥\left({ℵ}_{2}-{ℵ}_{1}\right)∥}_{\infty },$$

Where, $L\;{Ӽ}_{i}\left(g\right)$ is bounded linear operator.

Since all partial derivatives of ${Ӽ}_{i}$ are limited, then ∃ K>0, $\forall \;£\;({ℵ}_{1},{ℵ}_{2},g)$ is contain in $R^{4}.$

$$∥\sum_{i=1}^{4}L\;{Ӽ}_{i}\left(g\right)∥\leq K$$

(4.2) $$\Rightarrow ∥Ӽ\left({ℵ}_{2}\right)-Ӽ\left({ℵ}_{1}\right){∥}_{\infty }\leq K∥\left({ℵ}_{2}-{ℵ}_{1}\right){∥}_{\infty }$$

So, Ӽ is Lipschitz continuous in ℵ.

Theorem 4.2 If Ӽ(0,ℵ(0))=0 and $K(P^{*}\frac{1}{\Gamma (\xi )})<1,\;$then ∃ a unique solution of the system (5.1).

Proof Let Ӽ(0,ℵ(0))=0. We must demonstrate that ℵ(t) only satisfies system (3.3) if it satisfies the requirement.

(4.3) $$ℵ\left(t\right)=\;{\;}^{c}I_{t}^{\xi }Ӽ\left(t,ℵ\left(t\right)\right)$$

Suppose ℵ(t) satisfies system (3.3), applying the Caputo fractional-integral on Eq. (3.3),

$${\;}^{c}cI_{t}^{\xi }[{}^{FFM}D_{t}^{\xi }ℵ\left(t\right)]{=}^{c}I_{t}^{\xi }\left[Ӽ\left(t,ℵ\left(t\right)\right)\right]$$

We get,

(4.4) $$ℵ\left(t\right)=ℵ\left(0\right)+\frac{1}{\Gamma \left(\xi \right)}\int_{0}^{t}{\left(t-\Phi \right)}^{\xi -1}Ӽ\left(\Phi ,ℵ\left(\Phi \right)\right)d\Phi ,$$

∵ Ӽ(0,ℵ(0))=0 and $א\left(0\right)={א}_{0}$

So, Eq. (4.3) is satisfied.

Conversely, let ℵ(t) satisfies (4.3) then by condition Ӽ(0,ℵ(0))=0, consequently $א\left(0\right)={א}_{0}$

Hence the solution is satisfying the initial data.

Now, we have to show that the system has a unique solution.

Assume ƞ=(0,M) and $Q:C\left(ƞ,R^{4}\right)\to C\left(ƞ,R^{4}\right)$ is given as

$$Q\left[ℵ\left(t\right)\right]=ℵ\left(0\right)+\frac{1}{\Gamma \left(\xi \right)}\int_{0}^{t}{\left(t-\Phi \right)}^{\xi -1}Ӽ\left(\Phi ,ℵ\left(\Phi \right)\right)d\Phi ,$$

Eq. (4.4) implies,

(4.5) Q[ℵ(t)]=ℵ(t)

And sup norm on ƞ, $∥.{∥}_{ƞ}$ is

$$∥ℵ\left(t\right){∥}_{ƞ}=sup_{t\in ƞ}∥ℵ\left(t\right)∥,$$

Which shows a Banach space constructed by $C(ƞ,R^{4})$ and $∥.{∥}_{ƞ}.$

Also let the function μ:C[0,M]→C[0,M], which is defined by

μω=v

$$∵\;v=v\left(t\right)=\int_{0}^{t}k\left(t,\Phi \right)ℵ\left(\Phi \right)d\Phi ,$$

Where k(t,Φ):ƞ×ƞ→R is the kernal of μ is continuous on closed region ǝ=ƞ×ƞ.

So, it is bounded. Then ∃ $k_{0}\in R$, such that

$$\left|k\left(t,\Phi \right)\right|\leq k_{0},\;k\left(t,\Phi \right)\in \;ƞ\times ƞ,$$

And

$$sup_{t,\Phi \in ƞ}\left|k\left(t,\Phi \right)\right|\leq k_{0}$$

Also, we have to show that integral operator μ is bounded. Let,

$$∥\mu \omega {∥}_{ƞ}={∥\int_{0}^{t}k\left(t,\Phi \right)ℵ\left(\Phi \right)d\Phi ∥}_{ƞ}$$

∴ℵ(Φ) and k(t,Φ) are continuous therefore,

(4.6) $${∥\int_{0}^{t}k\left(t,\Phi \right)ℵ\left(\Phi \right)d\Phi ∥}_{ƞ}\leq \;O^{*}∥k\left(t,\Phi \right){∥}_{ƞ}∥ℵ\left(t\right){∥}_{ƞ}$$

With ℵ(t)∈ $C(ƞ,R^{4}),$ k(t,Φ)∈ $C({ƞ}^{2},R^{4})$ such that:

$$∥k\left(t,\Phi \right){∥}_{ƞ}=sup_{t,\Phi \in ƞ}\left|k\left(t,\Phi \right)\right|,$$

From (4.5)

$$∥Q\left[{ℵ}_{1}\left(t\right)\right]-Q\left[{ℵ}_{2}\left(t\right)\right]{∥}_{ƞ}\leq ∥\frac{1}{\Gamma \left(\xi \right)}\int_{0}^{t}\left({\left(t-\Phi \right)}^{\xi -1}(Ӽ\left(t,{ℵ}_{1}\left(t\right)\right)-Ӽ\left(t,{ℵ}_{2}\left(t\right)\right)\right)d\Phi ,∥$$

Applying (4.2) with result in (4.6), we have

$$∥Q\left[{ℵ}_{1}\left(t\right)\right]-Q\left[{ℵ}_{2}\left(t\right)\right]{∥}_{ƞ}\leq K\left(P^{*}\frac{1}{\Gamma \left(\xi \right)}\right)∥{ℵ}_{1}\left(t\right)-\;{ℵ}_{2}\left(t\right){∥}_{ƞ}$$

So, Q will therefore be the contraction if

$$K\left(P^{*}\frac{1}{\Gamma \left(\xi \right)}\right)<1$$

Hence because of the Banach space contraction principle, the system (3.2) has a unique solution.

## Stability Analysis of Model

In this section, we prove the stability of the fractional model. Various stability classes have been used to study the English model. Recently, several researchers have employed HU-type stability for simulations since it provides an approximation answer for difficult issues. for additional information regarding HU stability. To simplify, we first demonstrate the HU stability of model (3.2).

Definition 5.1 In the case when *_i,_* ${ℵ}_{i}$, i=1,2,3,4 and for all

(5.1) $$\left(S^{**},C^{**},\;T^{**},F^{**}\right)\in ℘,$$

Satisfying below inequality

(5.2) $$\begin{array}{c} \begin{array}{c} ∥S^{**}\left(t\right)-G_{1}\left(t,\;S\left(t\right)\right)∥\leq 1\\ ∥C^{**}\left(t\right)-G_{2}\left(t,\;C\left(t\right)\right)∥\leq 1 \end{array}\\ \begin{array}{c} ∥T^{**}\left(t\right)-G_{3}\left(t,\;T\left(t\right)\right)∥\leq 1\\ ∥F^{**}\left(t\right)-G_{4}\left(t,\;F\left(t\right)\right)∥\leq 1 \end{array} \end{array},$$

Such that for any (S,C,T,F)∈℘, model (3) is satisfied with the inequality below

(5.3) $$\begin{array}{c} \begin{array}{c} ∥S^{**}\left(t\right)-G_{1}\left(t,\;S\left(t\right)\right)∥\leq_{1}{ℵ}_{1},\\ ∥C^{**}\left(t\right)-G_{2}\left(t,\;C\left(t\right)\right)∥\leq_{2}{ℵ}_{2}, \end{array}\\ \begin{array}{c} ∥T^{**}\left(t\right)-G_{3}\left(t,\;T\left(t\right)\right)∥\leq_{3}{ℵ}_{3},\\ ∥F^{**}\left(t\right)-G_{4}\left(t,\;F\left(t\right)\right)∥\leq_{4}{ℵ}_{4} \end{array} \end{array}$$

Remark 5.2 If and only if there exists Ni∈M([0,Y],R) for any t∈[0,Y], then |Ni(t)|<I and the

$$\left(S^{**},C^{**},T^{**},F^{**}\right)$$

(5.4) $$\begin{array}{c} \begin{array}{c} S^{**}\left(t\right)=G_{1}\left(t,\;S\left(t\right)\right)+N_{1}\left(t\right),\\ C^{**}\left(t\right)=G_{2}\left(t,\;C\left(t\right)\right)+N_{2}\left(t\right), \end{array}\\ \begin{array}{c} T^{**}\left(t\right)=G_{3}\left(t,\;T\left(t\right)\right)+N_{3}\left(t\right),\\ F^{**}\left(t\right)=G_{4}\left(t,\;F\left(t\right)\right)+N_{4}\left(t\right) \end{array} \end{array}$$

Lemma 5.3 Assume that for every ${\;}_{i}>0$, $\left(S^{**},C^{**},T^{**},F^{**}\right)$ ∈℘ is a solution of Eq. (5.2). The functions $\left(S^{**},C^{**},T^{**},F^{**}\right)$ ∈H then the system will satisfy

(5.5) $$\begin{array}{c} \begin{array}{c} S^{**}\left(t\right)-\left\{S\left(0\right)+\frac{G_{1}\left(t,\;S\left(t\right)\right)\left(1-\xi \right)}{H\left(\xi \right)}\right\}+\frac{\xi }{H\left(\xi \Gamma \left(\xi \right)\right)}\int_{0}^{t}{\left(t-k\right)}^{\xi -1}G_{1}\left(k,\;S\left(k\right)\right)dk,\\ C^{**}\left(t\right)-\left\{C\left(0\right)+\frac{G_{2}\left(t,\;C\left(t\right)\right)\left(1-\xi \right)}{H\left(\xi \right)}\right\}+\frac{\xi }{H\left(\xi \Gamma \left(\xi \right)\right)}\int_{0}^{t}{\left(t-k\right)}^{\xi -1}G_{2}\left(k,\;S\left(k\right)\right)dk, \end{array}\\ \begin{array}{c} T^{**}\left(t\right)-\left\{T\left(0\right)+\frac{G_{3}\left(t,\;T\left(t\right)\right)\left(1-\xi \right)}{H\left(\xi \right)}\right\}+\frac{\xi }{H\left(\xi \Gamma \left(\xi \right)\right)}\int_{0}^{t}{\left(t-k\right)}^{\xi -1}G_{3}\left(k,\;S\left(k\right)\right)dk,\\ F^{**}\left(t\right)-\left\{F\left(0\right)+\frac{G_{4}\left(t,\;F\left(t\right)\right)\left(1-\xi \right)}{H\left(\xi \right)}\right\}+\frac{\xi }{H\left(\xi \Gamma \left(\xi \right)\right)}\int_{0}^{t}{\left(t-k\right)}^{\xi -1}G_{4}\left(k,\;S\left(k\right)\right)dk \end{array} \end{array}$$

(5.6) $$\begin{array}{c} \begin{array}{c} \leq {\left\{\frac{\left(1-\xi \right)}{H\left(\gamma^{\xi }\right)}+\frac{\xi }{H\left(\xi \right)\Gamma \left(\xi \right)}\right\}}_{1},\\ \leq {\left\{\frac{\left(1-\xi \right)}{H\left(\gamma^{\xi }\right)}+\frac{\xi }{H\left(\xi \right)\Gamma \left(\xi \right)}\right\}}_{2}, \end{array}\\ \begin{array}{c} \leq {\left\{\frac{\left(1-\xi \right)}{H\left(\gamma^{\xi }\right)}+\frac{\xi }{H\left(\xi \right)\Gamma \left(\xi \right)}\right\}}_{3},\\ \leq {\left\{\frac{\left(1-\xi \right)}{H\left(\gamma^{\xi }\right)}+\frac{\xi }{H\left(\xi \right)\Gamma \left(\xi \right)}\right\}}_{4} \end{array} \end{array}$$

Since our model satisfies existence and uniqueness than our suggested model (3.2) is HU-stable in a way that by using above lemma and remarks.

$$\left\{\frac{1-\xi }{H\left(\xi \right)}+\frac{\gamma^{\xi }}{H\left(\xi \right)\Gamma \left(\xi \right)}\right\}\Xi_{i}<1$$

Where $\Xi_{1}>0,\;i=1,\;2,\;3,\;4$ is a Lipschitz constant.

## FDE Stochastic Analyzer Based on Grunwald–Letnikov

The Grünwald–Letnikov (G-L) approach is repeated repeatedly, but the strategy's sum increases prolonged, reflecting the drawback. The factors c v are comprehensive approaches and have improving service features, such as being positive and having a large attenuation effect. As a result, they infer that the scheme has seamless features, although the corrective term produces some disruption. In demonstrations, a discrete variant of the Gronwall argument is highly useful. The robustness and erroneous predictions connected to linear test equations are investigated using the G-L approximation like a numerical technique.

Consider generic version of an FDE and its initial circumstances to illustrate the numerical results for FDEs relying on G-L:

(6.1) $$\begin{array}{c} {}_{\alpha^{\prime }}^{.}D_{t}^{\beta }=f\left(r,s\left(t\right)\right),\\ s^{\left(i\right)}\left(0\right)=s_{0}^{\left(i\right)},i=0,1,....n-1, \end{array}$$

Ivo Petras provided a detailed final cyclical prescription of a GL-based solution obtained from Equation (i):

(6.2) $$\frac{1}{h^{\beta }}\sum_{o=0}^{\left[\left(r-a\right)*1/h\right]}{\left(-1\right)}^{o}s\left(r-oh\right)\left(\begin{array}{c} \beta \\ o \end{array}\right)\approx f\left(s\left(t\right)t\right),$$

To summarize the aforementioned relationship, we obtain

(6.3) $$\sum_{o=0}^{\left[\left(r-a\right)*1/h\right]}{\left(-1\right)}^{o}s\left(r-oh\right)\left(\begin{array}{c} \beta \\ o \end{array}\right)+r\left(t\right)\approx h^{-\beta }f\left(r,s\left(t\right)\right),$$

In the form of nonlinear input grid systems, the interval tϵ[0,T]=[0,h,2h,….,Kh=T], where h is the step size indicator, $\left[0,T\right]=t_{0}=0,t_{1},\ldots ,t_{K}=T$, as well as any system to collect in the interval were also depicted as $t_{k}=kh$ for k=0,1,2...K. The aforementioned equation is represented as follows in discrete form:

(6.4) $$\sum_{o=1}^{k}{\left(-1\right)}^{o}\left(\begin{array}{c} \beta \\ o \end{array}\right)s\left(r_{k}-oh\right)+s\left(r_{k}\right)=h^{-\beta }f\left(r_{k},s\left(r_{k}\right)\right),k=0,1,2,....,K,$$

In simple usage, the above term is written as:

(6.5) $$\sum_{o=1}^{k}c_{0}^{\beta }y\left(t_{k}-ho\right)+y\left(r_{k}\right)=h^{-\beta }f\left(r_{k},s\left(r_{k}\right)\right),k=0,1,2,....,K,$$

Where ${c_{o}}^{\beta }$ is defined as:

(6.6) $${c_{o}}^{\beta }=\left(\begin{array}{c} \beta \\ o \end{array}\right){\left(-1\right)}^{o},$$

Or equivalently with ${c_{o}}^{\beta }$=1,

(6.7) $${c_{o}}^{\beta }=\left(1-\frac{1+\beta }{o}\right){c_{{}_{o-1}}}^{\beta },o=0,1,...,$$

The recursive form of the GL numerical solver is:

(6.8) $$s\left(r_{k}\right)=-\sum_{o=1}^{k}c_{o}^{\beta }y\left(r_{k-o}\right)+h^{-\beta }f\left(r_{k},s\left(r_{k}\right)\right),k=0,1,2,...,k.$$

**Proposed Methodology:**

This research uses the Caputo discrete fractional model to model English language learning, using Bayesian Regularization Artificial Neural Networks (BRA-NNs) for optimization and forecasting. The methodology involves the following major steps:

a) Problem Formulation:
  • The fractional-order model is modified to represent the dynamics of language learning.
  • A mathematical framework is developed, specifying state variables and governing equations.
b) Model Implementation:
  • The Caputo discrete fractional model is used to model language learning progress.
  • Numerical discretization methods are utilized to solve the governing equations.
c) Neural Network Integration:
  • BRA-NNs are trained with synthetic and real data regarding English language proficiency.
  • The network is tuned for accuracy with Bayesian regularization to avoid overfitting.
d) Validation and Evaluation:
  • The model is validated via numerical simulations, comparing predicted learning curves with theoretical predictions.
  • Metrics such as Mean Squared Error (MSE) and correlation coefficients are employed for evaluation.
e) Comparative Analysis
  • Results are contrasted with standard linguistic learning models to determine efficiency and applicability.
  • Scalability and computational feasibility are examined for wider implementation.
f) Limitations:
  • Limitations identified, such as computational constraints and absence of empirical validation, are addressed.

**Fig. 1:**
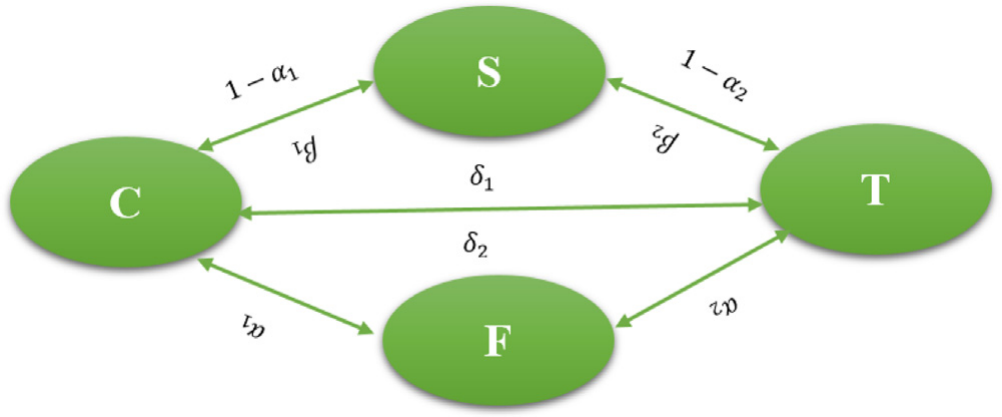
English language mathematical model’s block diagram.

To improve the reproducibility and readability of the computational method, we provide Algorithm 1 below the pseudocode describing the main steps of the suggested BRA-NNs-based simulation framework for the Caputo discrete fractional English language learning model is given in Fig. 1

**Algorithm 1:** BRA-NNs-based simulation of caputo discrete fractional english language model.

**Input:** - Initial population values: S₀, C₀, T₀, E₀ - Model parameters (μ, α₁, α₂, β₁, β₂, θ₁, θ₂, δ₁, δ₂) - Set Fractional order - Simulation time range [0, T] - Neural network hyperparameters (layers, learning rate, epochs, etc.) **Output:** - Simulated success probabilities for each fractional order - Performance metrics (MSE, regression R, gradient, etc.) - Comparison plots and absolute error analysis Step 1: Data Initialization - Set up model compartments: Susceptible (S), Modern Learners (C), Traditional Learners (T), Educated (E) - Define transition probabilities from Table 1 - Initialize time vector t ← [0, T] with step h Step 2: Generate Dataset For each fractional order γ in Ω: - Discretize Caputo derivative using GL or numerical approximation - Solve model Eqs. (3.2) using numerical method - Calculate Success Probability P_success = θ₁·T(t) + θ₂·C(t) - Store input-output pair: [S(t), C(t), T(t)] → P_success Step 3: BRA-NNs Training - Normalize input features and target values - Split data: 90 % training, 5 % validation, 5 % testing - Configure BRA-NNs architecture with Bayesian Regularization - Train network using MATLAB “nftool” or equivalent module - Record training metrics: MSE, gradient, mu, performance Step 4: Model Validation - Compare BRA-NNs predicted success probabilities with original model outputs - Compute Absolute Error (AE) for each fractional order - Plot: - MSE training curve - Regression (R) plot - Error histogram - AE vs. fractional order Step 5: Comparative Analysis - Implement Grunwald–Letnikov method for baseline comparison - Compare both models on MSE, AE, stability, and convergence - Tabulate and visualize differences End

**Fig. 2:**
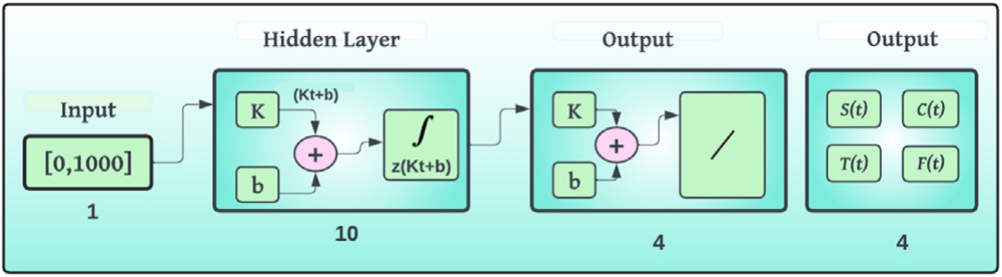
BRA-NNs topology.

**Fig. 3:**
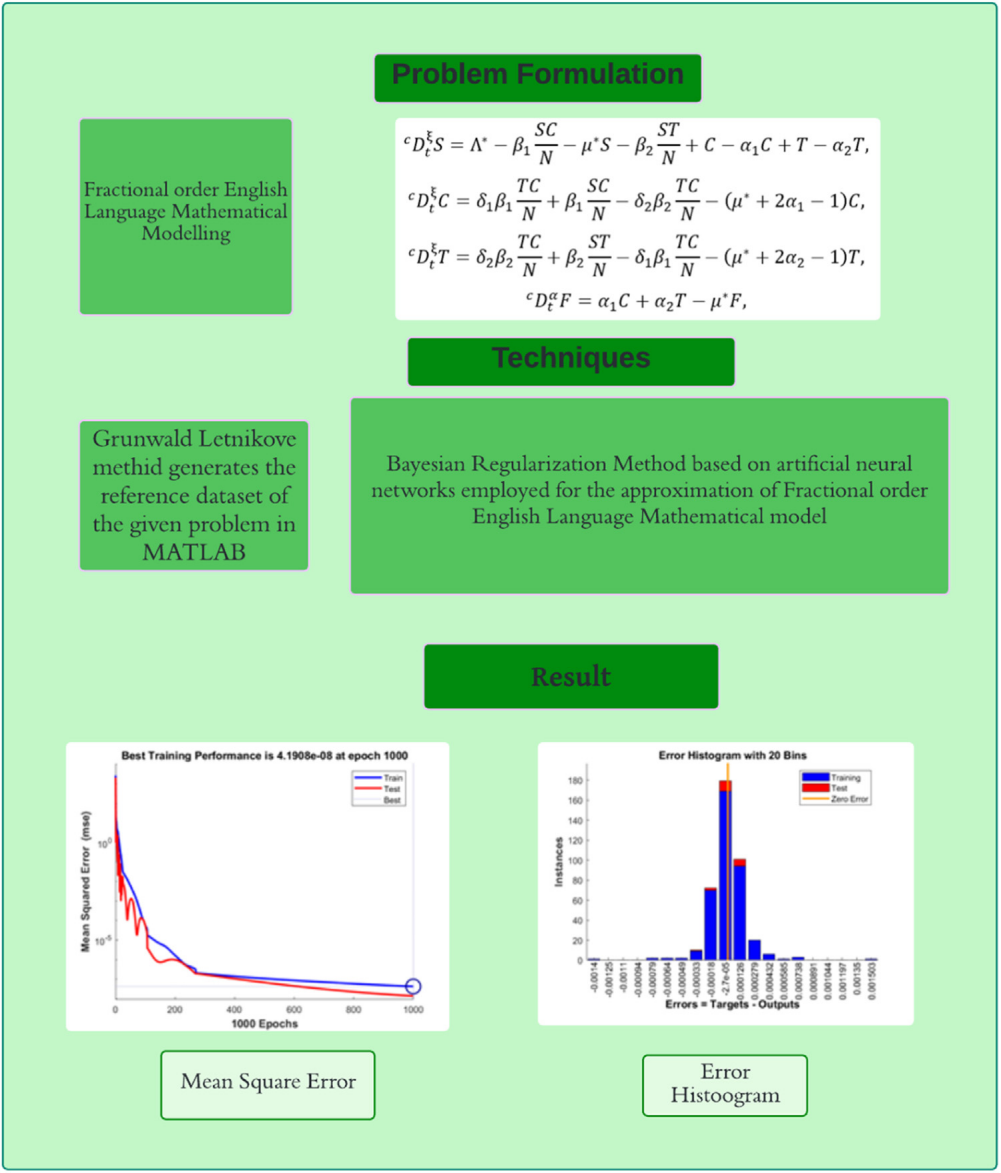
Graphical simulations of BRA-NNs for FOELMM.

An effective analogue to such a hypothetical neural network is a Bayesian regularized artificial neural network (BRA-NNs) with characteristic parameters that connect readily to physiochemical properties. The benefit of BRA-NNs would be that the predictions are resilient, and the recognition procedure, which in traditional regression approaches expands as $O(N^{2})$, is not required. Strength training is difficult because an evaluation approach gives an optimal threshold for terminating training and eliminates the requirement for a specific validation data to discover overtraining. As far as a minimum design is supplied, BRA-NNs are fundamentally agnostic to network design. The descriptors Bayesian and regularized must be properly specified, and in order to do so, basic regression procedures must be stated in Bayesian terms. Because vocabulary and representations differ from one approach to the next, creating concern, the representations included are uniform, but strange in certain cases. It is feasible to obtain at a stationary state instead of the universal minimal amount that's because the technique uses a gradient descent or comparable lowest reported. In comparison, with embodiment ANNs, hundreds or even thousands of repetition simulations are conceivable. The proposed BRA-NNs structure is used to address the FOELMM mathematical framework in this part. The strategy is divided into two sections. First, the fundamental BRA-NNs controller findings are shown. The BRA-NNs execution approach is also used to solve the FOELMM mathematical model. Fig. 2 depicts the BRA-NNs configuration, whereas Fig. 3 depicts the multi-layer proposed methodology employing numerical deterministic BRA-NNs. Fig. 2 proposed BRA-NNs model topology. The structure has an input layer, two hidden layers, and an output layer. Training is performed utilizing BRA (Balanced Random Adaptive) optimization such that the layer connections signify learned weights. The nodes in each of the two hidden layers make use of a non-linear activation function to map complex relationships between the dataset. This architecture allows the model to learn strong representations for prediction problems. The image has been resized and labeled with easier-to-read text and labels. The BRA-NNs processes are available in MATLAB via the “nftool” function, with the following data set up: 90% for training, 5% for testing, and 5% for permission. Table 5 details the simulations by BRA-NNs applied to FOELMM, for six cases with 1000 epochs of training. Six cases are shown, each using a different value of the fractional order, as 0.4, 0.5, 0.6, 0.7, 0.8, and 0.9, respectively; these values cover the progressive steps in the variations of the fractional order of the system. Each case is another simulation scenario wherein the fractional order directly influences the behavior of the system and consequently its stability, convergence, and performance. In the following table, it will be evident as the training outcome for every value of fractional order is included, with important key performance metrics like MSE in training and testing values, and estimates for gradient, mu, and performance. Such simulations are crucially important to point out the ability of the proposed BRA-NN model to generalize across all fractional orders where high adaptability and robustness towards complex, nonlinear dynamics associated with fractional-order systems are demonstrated.

**Table 5:** Simulations of BRA-NNs for FOELMM.

Case	Mean square error		Performance	Mu Parameter	Gradient	Time
	Training	Testing				
**1**	1.82E-007	7.98E-008	1.83E-007	500	3.32E-005	2
**2**	2.43E-007	1.62E-006	2.44E-007	50	6.84E-005	2
**3**	4.19E-008	1.37E-008	4.19E-008	500	0.000339	2
**4**	4.99E-008	3.10E-008	5.00E-008	500	4.47E-005	2
**5**	1.65E-008	1.10E-008	1.66E-008	500	6.51E-005	2
**6**	7.67E-008	1.10E-007	7.67E-008	50	0.000244	2

**Fig. 4:**
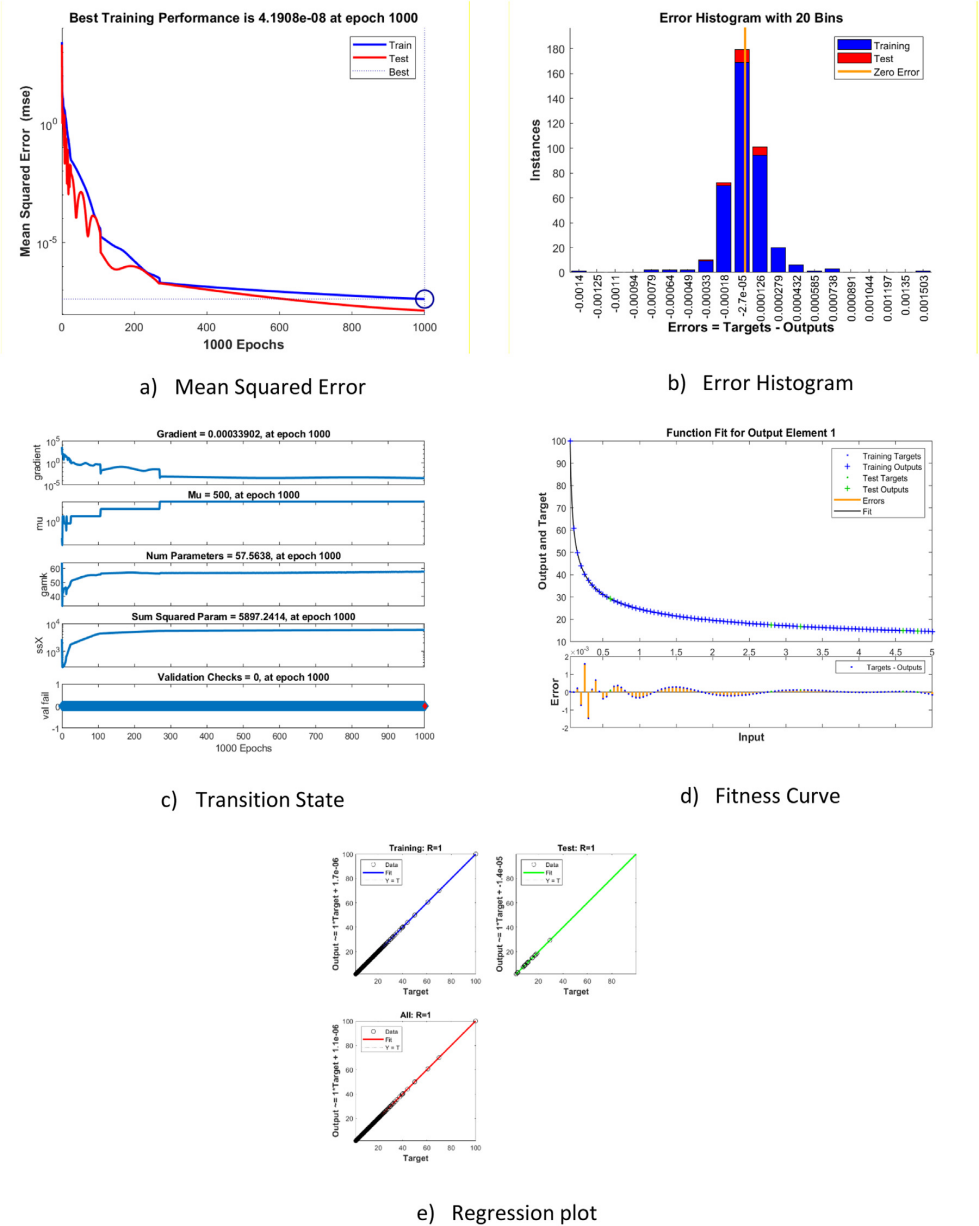
Statistical results of BRA-NNs for FOELMM for case 3.

Fig. 4 reports the schematic results that come with the results from BRA-NNs on case 3 concerning training performance. The plot for Fig. 4a depicts performance using the MSE accrued by the networks while it underwent the process of training up to a surprisingly minimal value of 4.19E-08 at epoch = 1000, as indicating that the developed model had significant accuracy. Fig. 4(c) shows the training state, with Case 3 having an estimated gradient and mu of 0.00033902 and 500, respectively, which means that the training is stable and that the network weights are handled well during optimization. In addition, the number of parameters (num parameter) and the sum of squared parameters are estimated to be 57.56 and 5897.24, respectively, which depicts the complexity of the network and utilization of parameters. Figs. 4(b), (d), and (e) plot some plots of how the network had performed. This error histogram in Fig. 4(b) shows that which the error values with which the fitted values are much closer to their target values. The BRA-NN's dependability is illustrated via the histogram. The histogram graph has twenty vertical bars, or bins. The vertical orange line that separates the data into positive and negative segments is known as the zero-error line. The link between the goal values and the output is graphically displayed by the regression function. The fitness curve in Fig. 4(d) shows that the model is optimized, and the regression analysis in Fig. 4(e) shows that the predicted and actual outputs are highly correlated, indicating that the BRA-NN model is robust for Case 3. A relationship with a R=1 is said to be linear. The optimum match is demonstrated by a straight line. The output equals the target (Y=T), as indicated by the dotted lines. All these plots validate the performance, accuracy, and reliability of the network in modeling the given dataset.

**Fig. 5:**
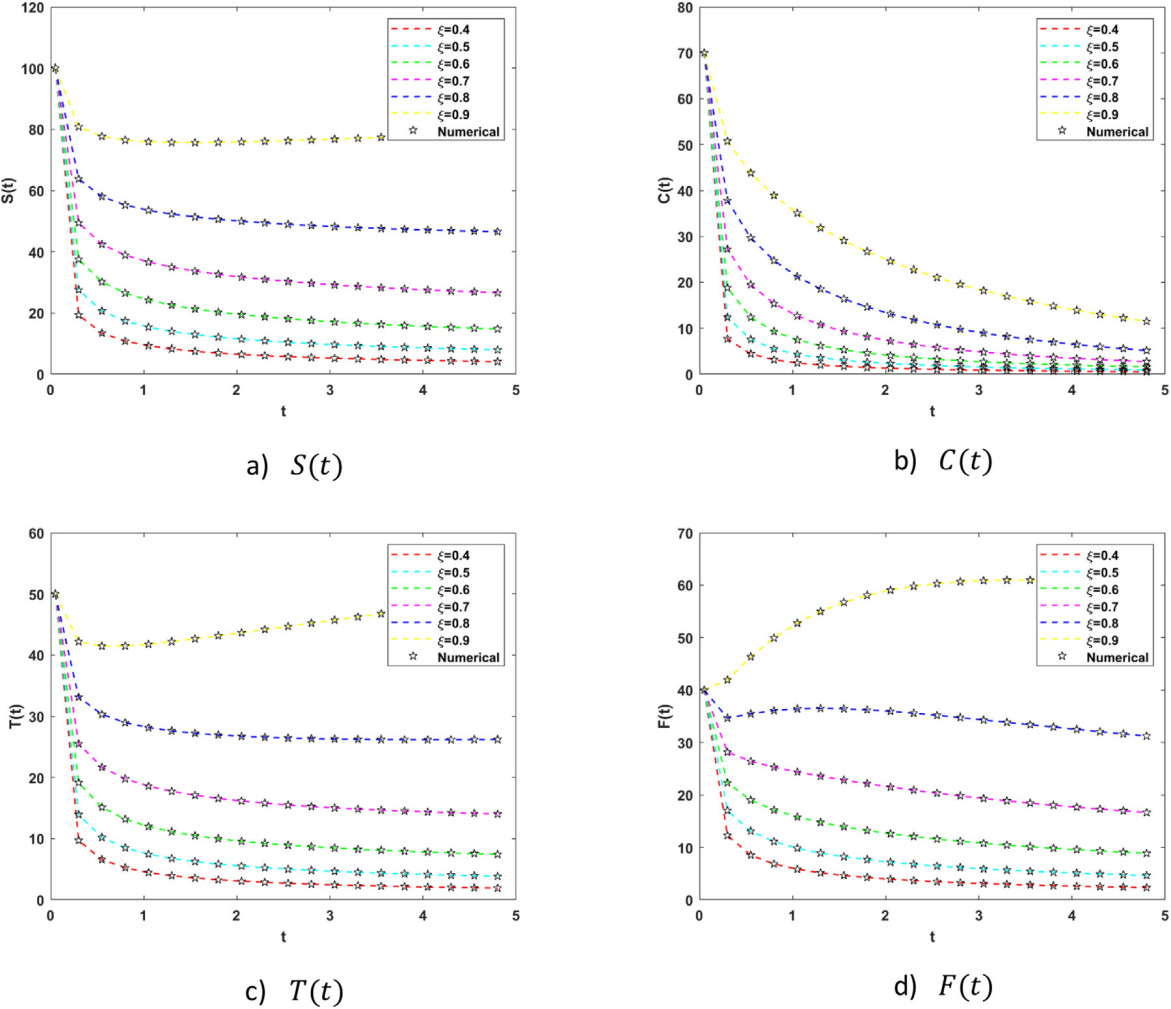
Comparative analysis plots of FOELMM using Grunwald Letnikov scheme and BRA-NNs.

**Fig. 6:**
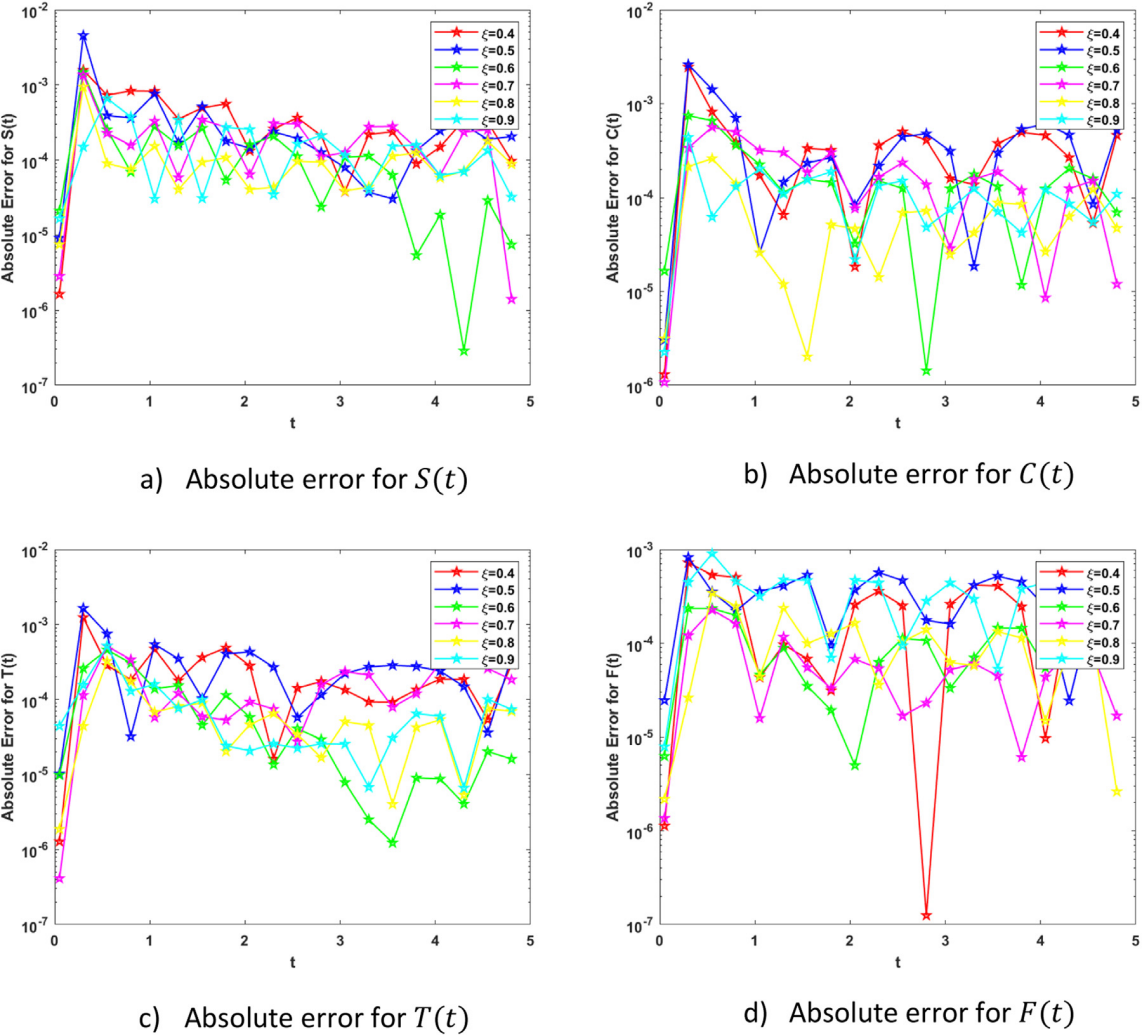
Absolute Error Analysis plots of FOELMM using Grunwald Letnikov scheme and BRA-NNs.

In particular, Fig. 5 show the results of S(t),C(t),T(t) and F(t) with parameters $\alpha_{1}$ represents the percentage of language learners who successfully complete a traditional course, $\alpha_{2}$the percentage of language learners who successfully complete a modern course, and $\beta_{1}$ and $\beta_{2}$ respectively represent the likelihood that people will use traditional education to learn English, and modern education, respectively. A student learning a language through traditional education switches to one learning it through modern education at $\delta_{1}$; similarly, a student learning a language through modern education switches to one learning a language through traditional education at $\delta_{2}$, $\mu^{*}\;$the rate of natural death in the population as a whole on the basis of six different fractional orders ξ=0.4,0.5,0.6,0.7,0.8,0.9. The Fig. 5(a-d) illustrates that by the increasing value of fractional order the S(t) the percentage of susceptible individuals who regularly attend language learning sessions, C(t) the percentage of individuals who choose to pursue modern education, T(t) the percentage of individuals who choose traditional education and F(t) the percentage of individuals who completed their course successfully got increased on the lowest fractional order it converged to 0 but with the increment of fractional order is went far from 0. At the highest fractional order, the percentage of individuals who completed their course on increased which shows the best result. Fig. 5 also contains the comparative results of numerical scheme with grunwald letnikov method. Fig. 6 shows absolute error plots with AE values ranging from $10^{-2}\to 10^{-7}$, $10^{-2}\to 10^{-6}$, $10^{-2}\to 10^{-7}$ and $10^{-3}\to 10^{-7}$ for S(t),C(t),T(t), and F(t) respectively which shows that BRA-NNs performs excellent with the comparison of grunwald lentikov method. Both Class T and Class C success rates are improved by raising the fractional order, as seen in Fig. 6. Under Class C dynamics, the transition probability $\delta_{1}=\;0.35$ leads to a notable rise of successful learners, highlighting the advantages of contemporary educational resources and methods.

**Method validation**

Numerical simulations and comparative analysis were used to validate the suggested Caputo discrete fractional model for English language learning. Several methods were used to evaluate the methodology to guarantee its robustness, accuracy, and dependability.

1. Numerical Simulations and Empirical Evidence
  • The effectiveness of the suggested model was validated by means of comprehensive numerical simulations.
  • The fractional-order English Language Mathematical Model (FOELMM) was divided into six fractional-order variations, which were then tested.
  • The theoretical conclusions were empirically supported by the simulation results.
2. Comparison with Benchmark Methods
  • By comparing the model to the Fractional-Order Lotka-Volterra approach, the model's accuracy was confirmed.
  • The outcomes validated the suggested randomized computational solver's (BRA-NNs) dependability.
3. Computational Solver Accuracy
  • Bayesian Regularization Artificial Neural Networks (BRA-NNs) were used to solve the model numerically, guaranteeing excellent precision.
  • The model's performance was maximized throughout the training phase, resulting in low Mean Squared Error (MSE) values.
  • The model's performance was maximized throughout the training phase, resulting in low Mean Squared Error (MSE) values.
  • Multiple training iterations confirmed the model’s convergence and stability.
4. Absolute Error Analysis
  • A comparison error study was conducted between BRA-NNs and the Grunwald-Letnikov technique.
  • The method's resilience was further validated by the absolute error staying within a predefined threshold.
5. Performance Metrics and Predictive Accuracy
  • The model's predictive power was validated using key validation measures such as MSE, regression results, and parameter estimations.
  • The projected values closely matched the actual results, according to graphic simulations.

The suggested model was shown to be a precise, consistent, and trustworthy method for examining language acquisition dynamics using various validation procedures.

**Limitations**

The limitations have been organized below:

• Computational Demand: The Caputo discrete fractional model, although numerically accurate and trustworthy, poses huge demands on computational power because of the Bayesian Regularization Artificial Neural Networks (BRA-NNs), which are intensive processors for training and optimization.
• Lack of Empirical Validation: The model validation is only through numerical simulations, and no empirical validation with human subjects.
• Limited Linguistic Applicability: The model has not been tested for its viability with varying languages or linguistic structures.
• Interpretability Issues: The “black-box” representation of Artificial Neural Networks presents interpretability issues, which could restrict its potential in the educational policy framework.
• Scalability Implications: More work is required to determine the model's scalability and reliability when tested for multiple cases (N cases) in practical applications.

**Future Direction**

As a future research direction, we plan to extend this work by:

• Putting into practice and examining fractional-order models with other memory kernels.
• Comparative simulation studies to determine the performance of such models with respect to our existing Caputo-based formulation.
• Exploring the appropriateness of various memory behaviors in representing the intricacies of long-term language acquisition and learning adaptation.

## CRediT authorship contribution statement

**Maria:** Conceptualization, Investigation, Software, Visualization, Writing – original draft. **Aqsa Zafar Abbasi:** Conceptualization, Investigation, Visualization, Writing – original draft. **Muhammad Asif Zahoor Raja:** Formal analysis, Methodology, Supervision, Validation, Writing – original draft. **Kottakkaran Sooppy Nisar:** Formal analysis, Software, Validation, Writing – original draft, Writing – review & editing. **Muhammad Shoaib:** Investigation, Software, Visualization, Writing – original draft.

## Declaration of competing interest

The authors declare that they have no known competing financial interests or personal relationships that could have appeared to influence the work reported in this paper.

## Data Availability

No data was used for the research described in the article.
